# Polycomb complexes in X chromosome inactivation

**DOI:** 10.1098/rstb.2017.0021

**Published:** 2017-09-25

**Authors:** Neil Brockdorff

**Affiliations:** Department of Biochemistry, University of Oxford, South Parks Road, Oxford OX1 3QU, UK

**Keywords:** X inactivation, *Xist* RNA, Polycomb

## Abstract

Identifying the critical RNA binding proteins (RBPs) that elicit *Xist* mediated silencing has been a key goal in X inactivation research. Early studies implicated the Polycomb proteins, a family of factors linked to one of two major multiprotein complexes, PRC1 and PRC2 (Wang 2001 *Nat. Genet.* **28**, 371–375 (doi:10.1038/ng574); Silva 2003 *Dev. Cell* **4**, 481–495 (doi:10.1016/S1534-5807(03)00068-6); de Napoles 2004 *Dev. Cell* **7**, 663–676 (doi:10.1016/j.devcel.2004.10.005); Plath 2003 *Science* **300**, 131–135 (doi:10.1126/science.1084274)). PRC1 and PRC2 complexes catalyse specific histone post-translational modifications (PTMs), ubiquitylation of histone H2A at position lysine 119 (H2AK119u1) and methylation of histone H3 at position lysine 27 (H3K27me3), respectively, and accordingly, these modifications are highly enriched over the length of the inactive X chromosome (Xi). A key study proposed that PRC2 subunits bind directly to *Xist* RNA A-repeat element, a region located at the 5′ end of the transcript known to be required for *Xist* mediated silencing (Zhao 2008 *Science* **322**, 750–756 (doi:10.1126/science.1163045)). Subsequent recruitment of PRC1 was assumed to occur via recognition of PRC2 mediated H3K27me3 by the CBX subunit of PRC1, as has been shown to be the case at other Polycomb target loci (Cao 2002 *Science* **298**, 1039–1043 (doi:10.1126/science.1076997)). More recently, several reports have questioned aspects of the prevailing view, both in relation to the mechanism for Polycomb recruitment by *Xist* RNA and the contribution of the Polycomb pathway to *Xist* mediated silencing. In this article I provide an overview of our recent progress towards resolving these discrepancies.

This article is part of the themed issue ‘X-chromosome inactivation: a tribute to Mary Lyon’.

## 1. Recruitment of PRC2 complexes by *Xist* RNA

**Figure 1.**
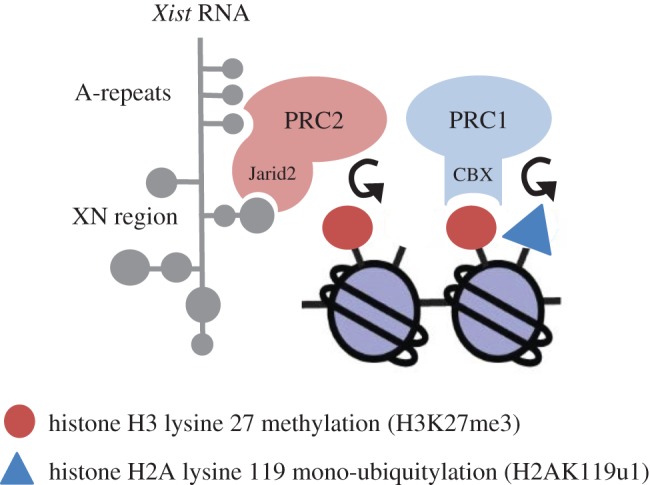
Classical model for Polycomb recruitment by *Xist* RNA. Early studies proposed direct interaction between core PRC2 subunits and the A-repeat element in *Xist* RNA. Subsequent studies implicated the *Xist* XN region and the PRC2 cofactor Jarid2 in initiating PRC2 recruitment. PRC2 functions to catalyse H3K27me3 on underlying nucleosomes. PRC1 recruitment is indicated as occurring downstream through interaction of the PRC1 subunit CBX and PRC2 mediated H3K27me3. Recruitment of PRC1 in turn mediates H2AK119u1 deposition on underlying chromatin.

Experiments on early mouse embryos and using inducible *Xist* transgenes in mouse embryonic stem cells (mESCs) revealed that Polycomb recruitment in X inactivation is strictly dependent on ongoing *Xist* RNA expression [1,2]. Thus, detectable enrichment of Polycomb complexes and associated modifications on the inactive X chromosome (Xi) occur rapidly as *Xist* RNA expression commences, and disappear when *Xist* transgene expression is extinguished. The requirement for ongoing *Xist* RNA expression has been interpreted to indicate that *Xist* RNA recruits Polycomb complexes either directly or through an intermediary RNA binding protein (RBP). In support of this view, conventional and super-resolution microscopy studies indicate that *Xist* RNA and Polycomb subunits localize closely with one another [3–5] (although see below), a conclusion that is further supported by high-throughput chromatin immunoprecipitation based approaches that report that Polycomb occupancy on Xi correlates strongly with sites of *Xist* RNA binding [4,6–10]. Critically, it has been reported that the PRC2 subunit Ezh2 binds *Xist* RNA (or a short isoform of *Xist* RNA, RepA) directly, via the A-repeat, an element at the 5′ end of the transcript that is critical for *Xist* mediated chromosome silencing [11]. This finding, based largely on *in vitro* interaction studies, established a model in which Polycomb recruitment to Xi is initiated by PRC2 binding to the *Xist* A-repeat. PRC1 recruitment was inferred to occur through the classical hierarchical pathway in which the CBX subunit present in canonical PRC1 complexes binds to PRC2 mediated H3K27me3 (figure 1). Some recent variations on this theme include the suggestion that the PRC2 subunit Suz12 mediates the interaction with *Xist* RNA [12], that Ezh2 phosphorylation is required for efficient RNA binding [13], and more recently, that a PRC2 cofactor, Jarid2, mediates binding to *Xist* RNA [14], although in the latter case through an element located downstream of the A-repeats (see below). It has also been reported that the chromatin remodeller ATRX facilitates loading of PRC2 onto the A-repeat element [15], although more recent work reported that *ATRX* knockout has no significant effect on PRC2 recruitment to Xi [16].

While the aforementioned model provides a plausible explanation for Polycomb recruitment by *Xist* RNA, there are several experimental observations that are difficult to reconcile. In early mouse embryos the onset of *Xist* RNA expression significantly precedes appearance of Polycomb enrichment on Xi, despite the continued presence of core PRC2 complexes [17]. In addition, evidence that the *Xist* A-repeat mediates interaction with PRC2 subunits is countered by the observation that *Xist* lacking the A-repeat is still able to recruit PRC2 [14,18]. It has been claimed that in this circumstance levels of PRC2 recruitment are significantly reduced [11], but others report little or no difference [14,16]. Given that immunofluorescence is not ideally suited to quantitative analysis, this question remains open. While the contribution of the A-repeat region in PRC2 recruitment is debatable, more recent work has pointed to a critical role for an element immediately downstream, within a 3–4 kb region, termed XN [14]. PRC2 recruitment via the XN element is mediated by the PRC2 cofactor, Jarid2.

A further consideration is that although microscopy studies point to there being a close association of *Xist* and PRC2, super-resolution three dimensional structured illumination microscopy (3D-SIM) indicates that the spatial separation is in fact is greater than would be expected for directly interacting factors [4]. This conclusion has been disputed in a subsequent study using a different super-resolution microscopy platform, PALM/STORM [5]. In this latter study the conditions used for sample preparation were relatively disruptive, and this could account for any reported differences.

A final argument is that proteomic analysis of the *Xist* interactome failed to identify core PRC2 subunits, although interestingly PRC1 proteins were detected [19,20]. In this regard it should also be noted that neither Ezh2 nor Suz12 have a domain resembling RNA binding domains found in other proteins. Indeed domains or residues in these proteins important for RNA binding have yet to be defined, with the possible exception of phosphoSer345 in Ezh2 [13]. A domain in Jarid2 has been proposed to mediate interaction with RNA [21] but deletion of this region has no effect on PRC2 recruitment by *Xist* RNA [14].

## 2. Recruitment of PRC1 complexes by *Xist* RNA

A further topic of debate has been the basis for recruitment of PRC1 to Xi. As indicated in §1, this was initially attributed to the classical hierarchical model for PRC1 recruitment [22]. However, Shoeftner *et al.* [23] found that PRC1 mediated H2AK119u1 is present on Xi in the absence of PRC2/H3K27me3, demonstrating an alternative pathway for PRC1 recruitment. In subsequent work it was reported that non-canonical PRC1 complexes, in which the CBX subunit is substituted by the protein RYBP/YAF2 [24,25], account for PRC2 independent recruitment of PRC1 to Xi and also to other sites in the genome [25]. These observations were initially interpreted to indicate the existence of separate pathways for *Xist* dependent recruitment of PRC2 and of non-canonical PRC1. However, an alternative interpretation came to light with reports demonstrating that PRC2 can recognize PRC1 mediated H2AK119u1 [26–28], referred to herein as reverse hierarchical recruitment. The discovery of the reverse pathway raises the intriguing possibility that PRC1 recruitment precedes that of PRC2 at sites genome wide, including Xi.

**Figure 2.**
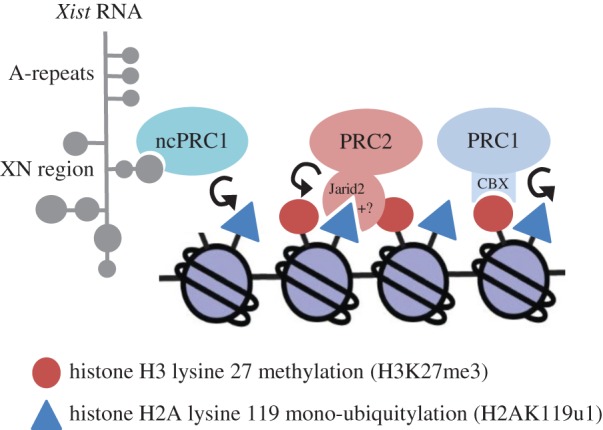
Revised model for Polycomb recruitment by *Xist* RNA. The Polycomb cascade is initiated by non-canonical (nc) PRC1 complexes that are recruited by the *Xist* XN region. PRC1 mediated H2AK119u1, deposited on underlying nucleosomes, serves to recruit PRC2 through recognition by the cofactor, Jarid2 or through an alternative but currently undefined pathway (+?). PRC2 mediated H3K27me3 then signals recruitment of canonical PRC1 complexes, further reinforcing H2AK119u1 deposition and Polycomb domain formation.

In recent work we set out to determine if Polycomb recruitment to Xi is initiated by PRC1, PRC2 or both complexes together. Interestingly we found that PRC2 recruitment is entirely dependent on prior deposition of H2AK119u1 by non-canonical PRC1 complexes [29]. Moreover, we found that recruitment of non-canonical PRC1 maps to the XN region of *Xist* RNA, previously implicated in PRC2 recruitment. Linked to these findings we have recently shown that Jarid2, the PRC2 cofactor implicated in interactions with the XN region [14], directly binds to H2AK119u1 through a ubiquitin interaction motif (UIM), located at the N-terminus of the protein [16]. Gene knockout of Jarid2 strongly abrogates PRC2 recruitment to Xi, although a low level of occupancy is retained, suggesting the existence of a secondary mechanism through which PRC2 recognizes H2AK119u1 by PRC2 [16]. A revised model for Polycomb recruitment by *Xist* RNA, based on the aforementioned results, is illustrated in figure 2.

## 3. The role of Polycomb complexes in chromosome silencing by *Xist* RNA

The first evidence linking the Polycomb system and X inactivation was the finding that embryos with a null mutation in the gene encoding the PRC2 subunit EED, display a female specific phenotype in which trophoblast giant cells (TGCs), an extraembryonic cell type found in the placenta and other extra-embryonic tissues, are largely absent [30]. This relatively subtle phenotype occurred together with phenotypes common to male and female Eed mutant embryos, notably a failure to gastrulate that results in embryonic lethality at approximately E7.5. The female specific effect on TGCs was linked to a failure in X inactivation through analysis of an X-linked GFP transgene. No effects were observed in embryonic or other extraembryonic lineages, although embryo lethality in Eed mutants at E7.5 limited the possibility to investigate this in depth. Indeed, following on from this seminal work, it was shown that Polycomb complexes are highly enriched on Xi, both in extraembryonic and embryonic cell types [3,31–33], suggesting a wider role in the X inactivation process. Along these lines, subsequent analysis of Eed mutant embryos using nascent RNA FISH to detect allelic expression of a small number of X-linked genes, indicated aberrant X inactivation in embryonic tissues [31]. Set against this, further studies failed to confirm this conclusion [34], and moreover reported that PRC2 is required to silence Xi genes only in differentiated extraembryonic lineages [35]. Support for a lineage specific role for PRC2 in Xi silencing in TGCs comes from a more recent study that demonstrates that TGCs are uniquely sensitive to Xi reactivation [36]. The implication here is that other silencing mechanisms, for example those mediated by the A-repeat element, function less well in TGCs, and as a consequence there is a unique dependence on the Polycomb pathway.

A second body of evidence indicating that Polycomb plays only a limited role in Xi gene silencing in embryonic lineages arises from studies in mESCs using inducible *Xist* transgenes. Deletion of the A-repeat element strongly abrogates silencing [18], yet recruitment of PRC1 and PRC2 is retained [2,14,16]. Moreover, deletion of other *Xist* RNA domains, including the XN region, was reported to have little or no effect on silencing [18]. Additionally, gene knockout of the PRC2 subunit Eed had no discernible effect on the ability of autosomally integrated *Xist* transgenes to silence genes located *in cis* [23]. Similarly, gene knockout of Ring1B, the principal catalytic subunit of PRC1, was found not to affect *Xist* mediated silencing [37].

While the aforementioned experiments indicate that Polycomb has at most a marginal effect on *Xist* mediated silencing in embryonic cells, there are some caveats that need to be considered. The assay to determine the silencing function of different *Xist* regions is relatively crude, relying on the occurrence (or not) of significant levels of cell death following induction of *Xist* expression from the single X chromosome in XY mESCs [18]. The absence of obvious silencing defects in the PRC2 (Eed) deficient mESC model needs to be interpreted in light of the fact that PRC1 and H2AK119u1, which may contribute to silencing, are retained [23,25]. Additionally, in the PRC1 (Ring1B) deficient model, PRC2 mediated H3K27me3, and to some degree H2AK119u1, are still present [37], the latter presumably reflecting the activity of the Ring1B functional homologue, Ring1A. The relevance of this latter consideration is underscored by our recent findings demonstrating that in the complete absence of PRC1 activity (Ring1A+ Ring1B null), PRC2 recruitment by *Xist* RNA is entirely abolished [29]. Importantly, none of the prior studies have assessed whether *Xist* mediated silencing is affected by complete abrogation of both PRC1 and PRC2 recruitment. Are these caveats relevant? The answer it would appear is very much in the affirmative. In our recent work we found that in ncPRC1 knockout embryos in which recruitment of both PRC1 and PRC2 to Xi is abolished, there is female specific lethality at around E7.5–8.5. Male embryos were also affected but died at a later stage, around E10.5. Female lethality at E7.5–8.5 is unlikely to be attributable simply to effects on the TGC lineage, as the dependence on a functional placenta is apparent only from E9.5 onwards. Moreover, in equivalent mutant mESCs expressing an inducible *Xist* transgene, we observed significantly reduced silencing of most genes across the entire inactivated chromosome [29].

## 4. Concluding remarks

Our recent findings demonstrating that non-canonical PRC1 initiates Polycomb recruitment by *Xist* RNA overturns previous models based on initiation by PRC2. The classical model gained traction largely because of the reported biochemical interaction of PRC2 with A-repeat sequences *in vitro* [11]. A key issue that could have contributed to this misleading result is that interaction with A-repeat RNA was demonstrated using individual PRC2 subunits or partial PRC2 complexes. This can lead to artefacts because charged surfaces that are concealed in the context of the holocomplex, are exposed and thereby available to bind RNA in a non-specific manner. It should be noted that while high specificity interaction of PRC2 with the A-repeat can now be effectively ruled out, it remains possible that PRC2 interacts with RNA in a manner that is physiologically relevant. This idea has indeed been the subject of some recent studies [38–40].

As noted in §2, we find that non-canonical PRC1 complexes initiate Polycomb recruitment through interaction with a sequence element in the XN region of *Xist* RNA. A key area for future studies is to identify the critical element(s) within the XN region, and to define the biochemical interactions that recruit non-canonical PRC1. Here it should be noted that the interaction may be direct, with a non-canonical PRC1 subunit(s) binding the XN region, or alternatively, via an adaptor protein that binds both the XN region and PRC1 complexes. The latter possibility is more probable as known RNA binding domains are not present in core non-canonical PRC1 subunits. Moreover, non-canonical PRC1 subunits were identified in the *Xist* interactome determined using formaldehyde but not UV cross-linking [19,20], suggesting that their interaction with *Xist* RNA is more likely indirect, i.e. via an adaptor protein.

It will be important in the future to determine the degree to which the revised recruitment model can account for all of the experimental observations relating to Polycomb recruitment by *Xist* RNA. As discussed in §1, Polycomb recruitment lags significantly behind expression of *Xist* RNA in early mouse embryos, and the reason for this remains to be determined. Additionally, experiments using *Xist* transgenes revealed that inducing expression in differentiated ESCs does not lead to Polycomb recruitment. However, if the *Xist* transgene is expressed transiently prior to differentiation, Polycomb recruitment does occur following differentiation [2]. Again, the molecular basis for this observation remains to be determined. A final point of interest will be to determine if the pathway that recruits Polycomb to Xi is used by other non-coding RNAs to regulate targets elsewhere in the genome.

In relation to the contribution of Polycomb to *Xist* mediated silencing, our recent findings provide substantive evidence of the importance of Polycomb, not only in a specialized extraembryonic lineage, TGCs, but in all lineages derived from the early embryo. The fact that this was not detected previously is likely attributable to the fact that we used a relatively sensitive chromosome wide transcriptome assay, and also, that in our experiments both PRC1 and PRC2 activity is entirely abrogated.

An important question for the future is to understand how Polycomb facilitates *Xist* mediated silencing activity. There is little or no detectable silencing in the absence of the A-repeat, yet Polycomb recruitment occurs similarly to wild-type *Xist* RNA. This observation indicates that Polycomb functions downstream of, or somehow supports primary silencing by A-repeat binding factors. We envisage two possible scenarios: Polycomb modifications may serve to stabilize gene silencing established by A-repeat binding factors. Thus, aberrant silencing in the absence of Polycomb would represent a defect in maintenance of gene silencing. Alternatively, by affecting the compaction state of Xi chromatin, Polycomb may function to facilitate the spread of gene silencing mediated by the A-repeats. This latter idea is predicated on evidence that Polycomb proteins, through self-association, play an important role in higher order folding of the chromatin fibre [41–44]. While we cannot at present differentiate between these models, a possible clue lies in genome wide localization analysis of Polycomb. At conventional target genes Polycomb accumulates to high levels over the promoter, and there is evidence that Polycomb mediated histone modifications directly affect initiation and/or elongation by RNA polymerase II [45–47]. On Xi, Polycomb rather forms a thin blanket over genes and intergenic regions corresponding to chromosomal domains where *Xist* RNA accumulates [6,7,10]. These observations, notably the absence of Polycomb accumulation at promoters of Xi genes, is in our view more compatible with the second scenario, in which large chromosomal domains modified by Polycomb facilitate the *in cis* spreading of primary silencing mediated by the A-repeat binding factors.

## Data accessibility

This article has no additional data.
